# Geographic coverage of demographic surveillance systems for characterising the drivers of childhood mortality in sub-Saharan Africa

**DOI:** 10.1136/bmjgh-2017-000611

**Published:** 2018-04-09

**Authors:** C Edson Utazi, Sujit K Sahu, Peter M Atkinson, Natalia Tejedor-Garavito, Christopher T Lloyd, Andrew J Tatem

**Affiliations:** 1 WorldPop, Department of Geography and Environment, University of Southampton, Southampton, UK; 2 Southampton Statistical Sciences Research Institute, University of Southampton, Southampton, UK; 3 Faculty of Science and Technology, Lancaster University, Lancaster, UK; 4 GeoData, University of Southampton, Southampton, UK; 5 Flowminder Foundation, Stockholm, Sweden

**Keywords:** child health, health systems evaluation, geographic information systems

## Abstract

A major focus of international health and development goals is the reduction of mortality rates in children under 5 years of age. Achieving this requires understanding the drivers of mortality and how they vary geographically to facilitate the targeting and prioritisation of appropriate interventions. Much of our knowledge on the causes of, and trends in, childhood mortality come from longitudinal demographic surveillance sites, with a renewed focus recently on the establishment and growth of networks of sites from which standardised outputs can facilitate broader understanding of processes. To ensure that the collective outputs from surveillance sites can be used to derive a comprehensive understanding and monitoring system for driving policy on tackling childhood mortality, confidence is needed that existing and planned networks of sites are providing a reliable and representative picture of the geographical variation in factors associated with mortality. Here, we assembled subnational data on childhood mortality as well as key factors known to be associated with it from household surveys in 27 sub-Saharan African countries. We then mapped the locations of existing longitudinal demographic surveillance sites to assess the extent of current coverage of the range of factors, identifying where gaps exist. The results highlight regions with unique combinations of factors associated with childhood mortality that are poorly represented by the current distribution of sites, such as southern Mali, central Nigeria and southern Zambia. Finally, we determined where the establishment of new surveillance systems could improve coverage.

**Key questions**

**What is already known?**

- Health and Demographic Surveillance Systems (HDSS) play a key role in providing data to monitor trends and causes of under-5 mortality (U5M) in countries where censuses are often irregular and vital registration systems are weak.
- The establishment and growth of networks of HDSS sites has enabled patterns and inferences on U5M and other demographic and health indicators across wider areas to be explored and inferred.
- Logistical and operational constraints often determine the locations of HDSS sites, potentially leading to an incomplete, biased and unrepresentative picture within networks of the wide range of factors that affect child mortality; hence, for effective extrapolation of findings to unmonitored areas, there is a need to ensure that they are as representative of the range of underlying factors as possible.

**What are the new findings?**

- Across 27 sub-Saharan African countries, HDSS sites are highly geographically clustered, but represent the typologies of characteristics associated with U5M relatively well.
- Mapping the similarities between HDSS sites in terms of the demographic and health characteristics of their areas provides quantitative guidance on comparability and extrapolation of inferences between sites.
- Southern Mali, central Nigeria, Eastern Kenya, Southern Niger and parts of southern Zimbabwe are the regions with demographic and health characteristics associated with U5M that are most poorly represented by the existing set of HDSS sites within the 27-country study area.

**Key questions**

**What do the new findings imply?**

- With the collection of existing HDSS sites providing an incomplete picture of the range of factors associated with U5M, caution should be taken in extrapolating inferences to poorly represented areas.
- The geospatial approaches outlined here can provide quantitative guidance on where to locate new sites to improve the representativeness of key factors being monitored over wide areas.

## Introduction

It has been estimated that 5.9 million children under the age of 5 years died in 2015, mostly from preventable causes.^1^ In sub-Saharan Africa (SSA), deaths in children under 5 are 14 times more likely than in high-income regions of the world, and the region bears about half of the world’s under-5 mortality (U5M) with around 3 million deaths occurring in 2015.^2^ Intervention measures aimed at achieving Millennium Development Goal 4 contributed to reductions in childhood mortality from 91 deaths per 1000 live births in 1990 to 43 per 1000 in 2015 globally.^3–5^ This reduction was inadequate to achieve the target of reducing 1990 U5M levels by two-thirds in 2015, and in SSA progress in reducing mortality was deemed to be slow in many countries.^1^ The Sustainable Development Goals (SDGs) were established in 2015, aiming to reduce child mortality to <25 per 1000 live births in all countries by 2030.^6^

Robust and reliable ongoing measurement of childhood mortality is vital to achieving development goals, measuring progress towards them and understanding which interventions are effective.^7–9^ With civil and vital registration systems incomplete and weak in many countries across SSA, and censuses and national household surveys undertaken irregularly, Health and Demographic Surveillance Systems (HDSS) play a key role in providing data to monitor trends and the causes of U5M, both within countries and across regions.^10 11^ An HDSS is a data collection system that undertakes longitudinal follow-up of a well-defined cohort in a clearly delineated geographical area, measuring health and demographic indicators typically at intervals of 3–4 months. These indicators include crude and age-specific birth/fertility and death rates, migration and infant, child and U5M rates. This ongoing cohort surveillance of health and demography in low-income countries provides valuable information on data-poor regions and reliable evidence for health policy design.^12 13^ Many HDSS sites have formed networks to facilitate pooling of data and provide information on wide-area demographic patterns and their determinants or specific aspects of population health, including the International Network for the Demographic Evaluation of Populations and their Health (INDEPTH),^11^ the newly established Child Health and Mortality Prevention Surveillance (CHAMPS) network,^14^ the Pneumonia Etiology Research for Child Health (PERCH)^15^ sites and the Global Enteric Multicenter Study (GEMS)^16^ sites. Many HDSS sites are members of multiple of these networks, while some remain independent. HDSS sites belonging to the INDEPTH network provide all-age health and demographic data relating to the populations being monitored. In these sites, causes of deaths occurring outside health facilities are determined through verbal autopsy interviews.^11^ The CHAMPS network, however, focuses primarily on providing data on the causes of neonatal mortality and U5M using minimally invasive tissue sampling and other sources of information.^14^

Childhood mortality in SSA has been shown to vary substantially over both space and time.^17–21^ The factors driving these variations remain poorly understood due mainly to a lack of data, though a range of metrics have been shown to be strongly associated with U5M and suggested as the leading determinants.^17 22 23^ These can be categorised broadly into risk and protective factors. Preventable diseases such as malaria, diarrhoea and pneumonia^1 24^ are among the risk factors known to impact child mortality most strongly. Maternal risk factors such as short birth intervals increase the risk of mortality in children due to inadequate recovery from a previous birth and the older child competing for the mother’s lean resources with younger siblings.^8 17 25^ Poor sanitation practices (eg, open defecation) enhance the transmission of diseases such as diarrhoea, which is a major cause of U5M in SSA.^26^ Nutrition-related conditions, such as stunting, led to about 3.1 million deaths in children in 2011.^27^ Education of women is a significant determinant of child survival as this makes room for better health choices for children and better management of childhood diseases.^25 28–30^ Other important protective factors include vaccination against infectious diseases, access to a health facility and exclusive breast feeding.^22 31–33^ In addition to these risk and protective factors, geospatial socioeconomic, climatic and environmental metrics have been shown to be correlated with U5M.^17 18^

The spatial and temporal variations that exist in childhood mortality rates and their associations with key driving factors mean that to obtain a complete picture of the trends and drivers of U5M within countries and across wider regions, networks of HDSS sites need to ensure they are as representative of the range of factors that exist as possible. Such networks have tended to grow or be established with logistical and operational constraints in mind to ensure successful operation. Selection of site locations is usually based on considerations such as the need to monitor high-risk/mortality areas or areas where a dearth of reliable data is deemed to pose the greatest challenge. However, these can result in a biased and incomplete picture without the full range of socioeconomic, ecological, demographic, health and geographic characteristics that exist across the SSA region and influence childhood mortality, being covered.^34 35^ In quantifying and understanding the causes and trends in U5M both within a country and across SSA as whole, if the data used come from surveillance sites that are all established in similar types of settings, and miss key regions with unique characteristics and drivers of mortality, then inaccurate and incomplete estimates will likely be produced.

Here, we assess at subnational scales the coverage of existing HDSS sites across SSA against a set of key factors shown to be strongly associated with rates of childhood mortality.^23^ Previous studies have assessed the spatial representativeness of the INDEPTH HDSS network in terms of its coverage of broad environmental factors^34^ and both environmental and socioeconomic factors,^35^ but these have not focused specifically on factors associated with childhood mortality or included sites beyond the INDEPTH network. We assess the extent of current coverage of the range of key factors associated with U5M in 27 countries across SSA, identifying where gaps exist. We highlight subnational areas, countries and regions with unique combinations of factors associated with childhood mortality that are poorly represented by the current distribution of sites and map out where the establishment of new surveillance systems could improve coverage.

## Data and methods

### Subnational data on U5M and associated factors

**Figure 1:**
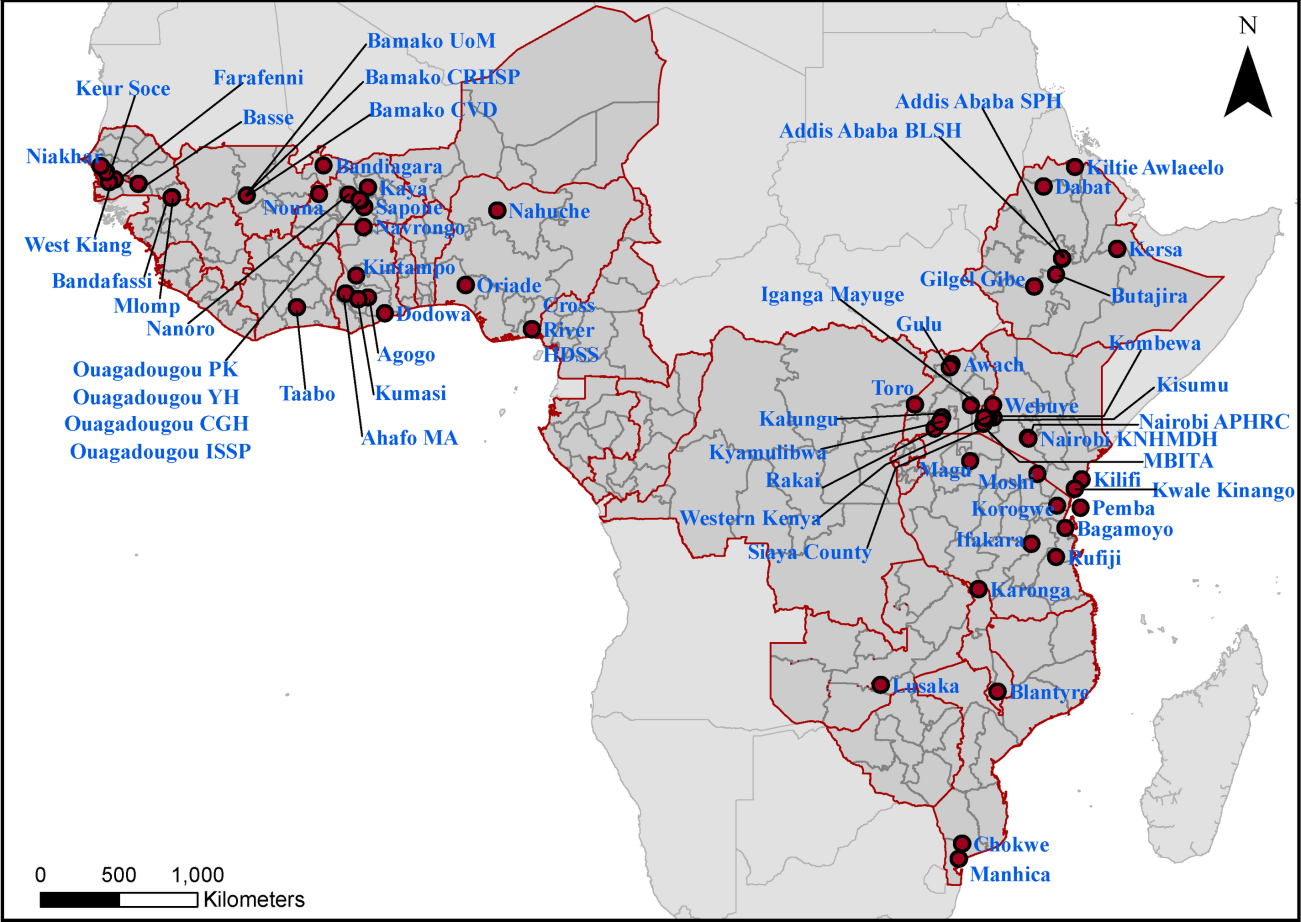
The subnational units for which under-5 mortality-associated variables were obtained, with the locations of the 65 Health and Demographic Surveillance Systems sites overlaid. Further details of the sites are provided in online supplementary table S1.

Following previous work,^23^ national household survey data from the 2010–2014 period covering 27 SSA countries were assembled from the Demographic and Household Surveys (DHS) programme.^36^ These covered a total of 255 subnational areas (figure 1). For each area, the seven factors found to be associated with U5M in Pezzulo *et al* 23 were calculated. These were six indicators derived from the survey data (female literacy, birth interval, access to a health facility, sanitation practices, vaccination coverage and stunting prevalence), as well as population-weighted *Plasmodium falciparum* malaria prevalence in children aged 2–10 years.^37^ Details of these variables are outlined in table 1, and their maps are shown in online supplementary materials.

10.1136/bmjgh-2017-000611.supp1 Supplementary data

**Table 1:** Demographic and Household Surveys and geospatial variables used in the study, following the work of Pezzulo *et al* 23

Variable	Description
Female literacy	Percentage of women between the age of 15 and 49 years who attended primary school and were able to read
Malaria prevalence	Proportion of children aged 2–10 years who had *Plasmodium falciparum* infection
Birth interval	Percentage of births with a birth interval of <36 months between birth and conception
Access to a health facility	Percentage of children born in the last five years preceding the survey who were delivered in a health facility (used as a proxy for access to a health facility)
Poor sanitation practices	Percentage of de jure population using fields, bushes, forests, open bodies of water or other open spaces rather than using the toilet to defecate
Vaccination coverage	Percentage of children aged 12–23 months who received measles vaccination at any time prior to the survey
Stunting prevalence	Percentage of children with a height that is at least 2 SD below the median height for children of the same sex and age

### Mapping HDSS sites

Through a survey of expert groups undertaken by programme officers at the Bill and Melinda Gates Foundation, and supplemented with literature and web searches, 65 active HDSS sites were identified within the 27 study countries. Thirty-one of these sites are part of the INDEPTH network,^11^ while the remaining sites are part of other networks, which include the PERCH^15^ and GEMS^16^ sites. These were georeferenced through assembling previous site location data,^34 35^ and additional sites were located through online gazetteers and maps. Figure 1 shows the location of the sites considered in these analyses, which fall into 46 of the 255 subnational units across the study area.

### Mapping the distribution of HDSS sites in relation to U5M factors

Variations exist in the driving factors behind U5M. To understand the potential of the existing set of HDSS sites in capturing the full range of variability in U5M and its associated factors, there is a need to quantify their coverage. To characterise the variations that exist in U5M-related factors across the 27 study countries, multivariate clustering of the factors across all 255 subnational units was undertaken. A previously developed Bayesian clustering approach was applied,^38^ and the cluster memberships were mapped and categorised by their distinguishing features (eg, relatively higher literacy rates and lower malaria prevalence than other clusters). Further details are provided in the online supplementary materials and Utazi *et al.* 38 The distribution of the HDSS sites across the identified clusters was then determined and plotted. In addition, the physical and demographic characteristics of the clusters, such as the total land area covered, average population density and total population covered, were also obtained for further evaluation of the clusters in terms of their representativeness across these different aspects. These analyses were undertaken in a Geographical Information System using population data from WorldPop^39^ (see http://www.worldpop.org).

### Measuring similarities between HDSS sites

Networks of surveillance sites aim to provide standardised and representative data on a broad range of health and demographic indicators. If the data and insights from these sites are to be compared, combined or used to make wider U5M inferences, there is a need to understand how similar they are in terms of factors associated with U5M and how representative they are of subnational areas without sites. To examine the similarities between the 65 HDSS sites in terms of the key indicators of U5M, a hierarchical clustering of the sites was undertaken. Following previous studies,^34 35^ similarity was measured through Euclidean distance in the multidimensional space defined by the key U5M indicators. Similarities were visualised through a complete-linkage dendrogram,^34 35^ with clear groupings of sites in distinct branches mapped.

### Mapping representativeness of U5M-associated factors by HDSS sites

To explore how representative the current set of HDSS sites are of the rest of the study region, Euclidean distances between the subnational areas where the sites are situated and each of the 255 subnational areas were calculated in the multidimensional space of the factors underlying U5M. These distances represented quantitative measures of similarity between each area containing a surveillance site and every other subnational area in the study region, in terms of the set of factors associated with U5M. There were 46 subnational areas hosting the sites, so this yielded a vector of 46 Euclidean distance values for each of the 255 subnational areas. The minimum Euclidean distance was determined for each subnational area and mapped to highlight how similar each area is to those areas with HDSS sites in terms of the factors associated with U5M. Large Euclidean distances are indicative of relatively poor representativeness by the current set of HDSS sites. This approach was also repeated with the individual indicators to determine the representativeness of the current range of HDSS sites of these indicators.

## Results

### HDSS site distributions in relation to U5M-related factors

**Figure 2:**
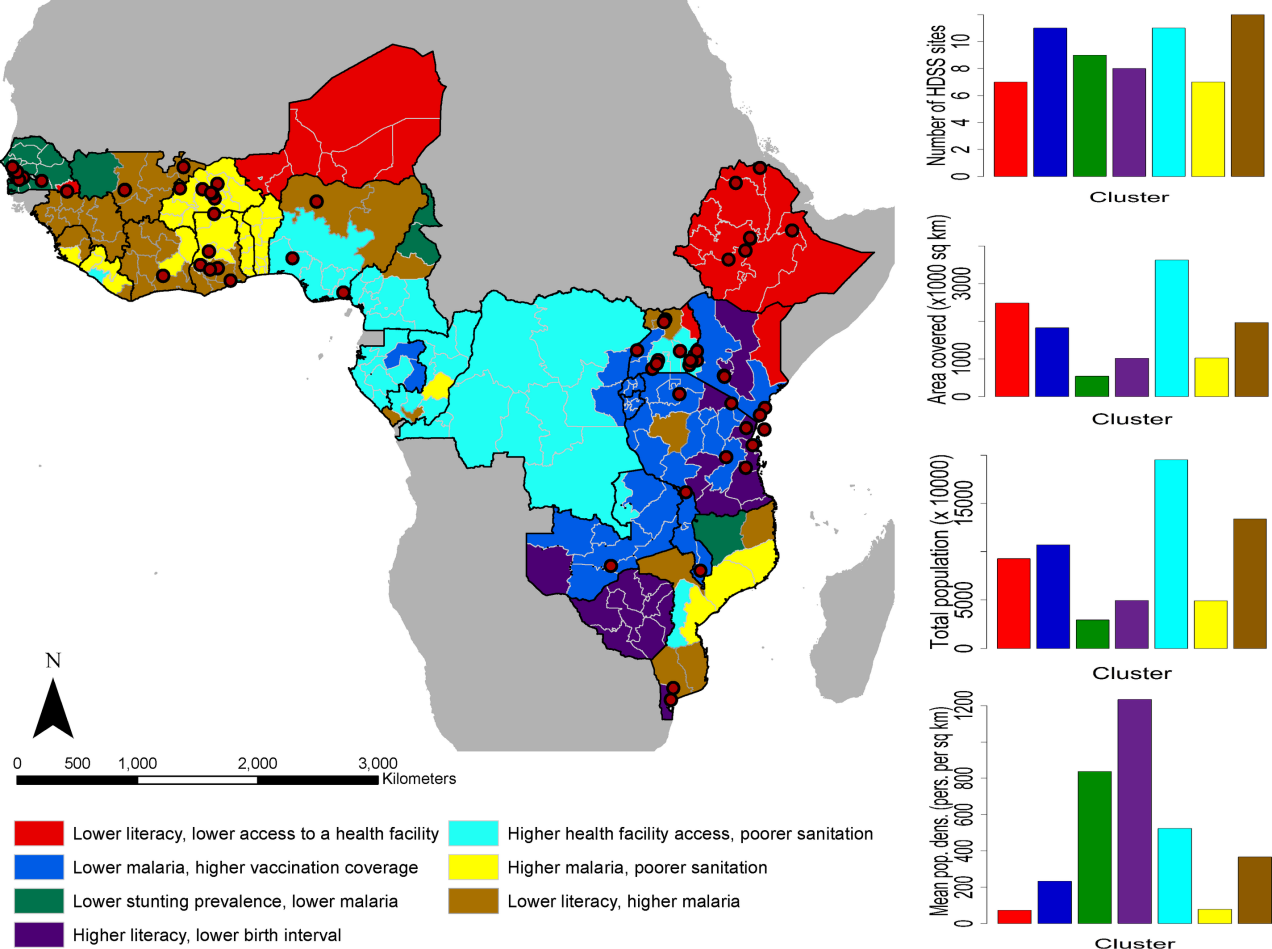
A cluster map showing geographical variation in under-5 mortality-related factors across the study region (left). The red-coloured dots are the locations of the Health and Demographic Surveillance Systems (HDSS) sites. At the right from top to bottom are the distribution of the HDSS sites across the clusters, total land area covered, total population and average population density of the clusters.

Substantial variation exists in the factors underlying U5M as shown by the map in figure 2, with seven groups of subnational areas identified across which mortality indicators are distinct. While sites are clustered geographically and large areas and entire countries remain without a site, the typologies of regions in terms of factors associated with U5M are relatively well-covered, as also shown by the plot in the top-right. The characteristics of the clusters based on these factors are shown in online supplementary figure S3. While indicators such as birth interval, vaccination coverage and stunting prevalence showed relatively low variation across the clusters, significantly greater variation in female literacy rates, malaria prevalence, access to a health facility and sanitation practices was seen.

Those regions with the combination of relatively higher rates of malaria and lower rates of literacy are represented by the largest number of sites, though these are mostly in West Africa. The regions with relatively higher health facility access but poorer sanitation levels are also represented by many sites, but these are largely within Uganda, and the extensive regions (figure 2, second graph down) that also fall into this typology in Democratic Republic of Congo, Cameroon and Nigeria are more poorly represented. In contrast, the class most poorly represented in terms of numbers of sites is characterised by relatively lower rates of literacy and health facility access. While it does overall cover the lowest mean population densities of all the typologies (figure 2, bottom graph), the total numbers of people remains high (figure 2, third graph).

The uncertainties (probabilities of being assigned to a cluster) associated with generating the cluster map in figure 2 are mapped in online supplementary figure S4. Subnational areas with high probabilities are strongly connected to their clusters, whereas areas with low probabilities have less affinity with their clusters. A very high proportion of the subnational areas were clustered with high probabilities which affirms the variation identified.

### Similarities between HDSS sites

**Figure 3:**
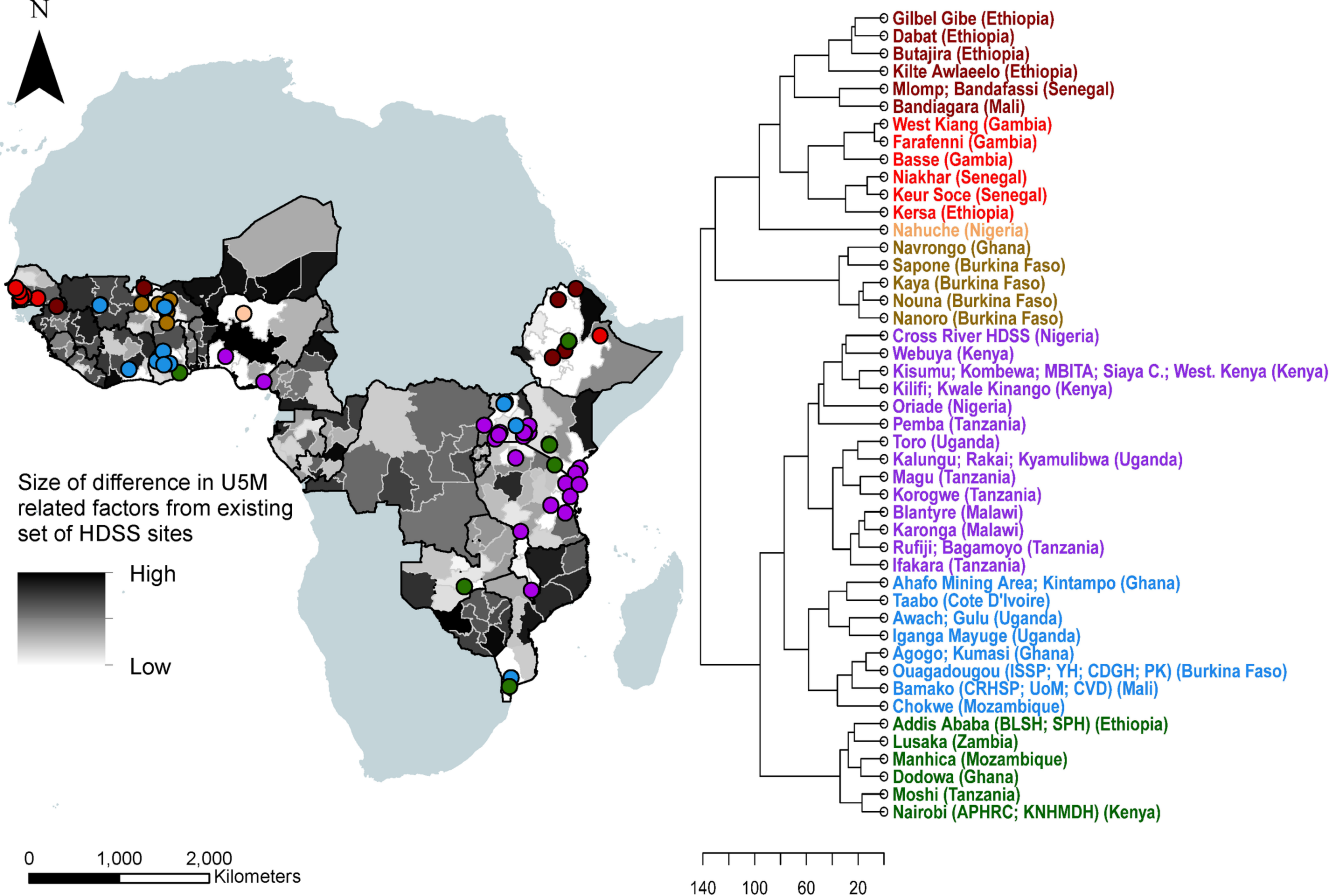
Minimum Euclidean distances between the Health and Demographic Surveillance Systems (HDSS) sites and the rest of the study area (left) and a dendrogram showing the similarities between the sites (right). The dots showing locations of the sites have the same colour scheme as the dendrogram. U5M, under-5 mortality.

Similarities between the HDSS sites in terms of factors associated with U5M are shown in figure 3, with subnational areas in the same class representing very similar sets of U5M-associated factors. For example, the class at the bottom of the dendrogram comprises sites in urban locations with relatively higher levels of the protective factors and lower levels of the risk factors (see online supplementary figure S5). There is a clear geographical clustering of many sites (eg, many sites in East Africa and West Africa are clustered together), although some geographically distant sites have similar features (eg, Cross River in Nigeria belongs to the major block of sites in East Africa, and Kersa in Ethiopia (East Africa) is clustered with sites in Senegal and Gambia (West Africa)). These classes of sites highlight how geography does not necessarily determine similarity in demographic and health factors. The main indicators that distinguish clearly the cluster with a single site—Nahuche (Nigeria)—from other clusters are poorer vaccination coverage and higher stunting prevalence (online supplementary figure S5).

### Representativeness of U5M-associated factors by HDSS sites

Areas most dissimilar from the current set of sites in terms of factors associated with U5M are highlighted in figure 3. These are poorly represented areas where U5M-related factors are very different from those where sites are located. Some subnational areas close to the sites are shown to be poorly represented which further suggests that proximity to sites does not necessarily equate to representativeness in terms of factors associated with and underlying U5M. Southern Mali, central Nigeria, Eastern Kenya, Southern Niger and parts of southern Zimbabwe are the most poorly represented areas and, therefore, most caution should be taken in extrapolating U5M inferences to these regions without new data. The map also serves as a guide to where the establishment of new sites could be prioritised for monitoring U5M to ensure better coverage of its associated factors, and a more complete understanding of its drivers across the continent.

## Discussion

In the era of the SDGs and their ‘leave no-one behind’ agenda, there is a need to ensure that data collection is as representative as possible of the range of heterogeneities that exist across and within low-income and middle-income settings. HDSS sites have played a key role in increasing our understanding of the dynamics and distributions of a wide range of demographic and health indicators, including U5M and its associated factors, but they represent an incomplete picture. Understanding how incomplete this is, where gaps exist and how to fill them is important if networks of HDSS sites are to be used to guide policies and strategies across national, regional and continental scales. Here, we have outlined approaches to assess the coverage of the collection of HDSS sites across multiple SSA countries and mapped the representativeness of existing site networks in characterising the drivers of U5M.

The results highlight that when combined together the suite of existing HDSS sites capture much of the variability in demographic and health factors related to U5M that exists across SSA. Unfortunately, while efforts to harmonise methods, share data and link sites together as networks are growing, some fragmentation exists, meaning that insights are rarely drawn from this full set of sites and the representativeness of individual site networks remains relevant.^35 38^ Newly established site networks, such as CHAMPS,^14^ cover a much less complete picture of the range of demographics, geographies and health patterns that exist, but are continuing to grow, and the approaches presented here can provide guidance on directions for expansion. The fact that geographical proximity to sites does not necessarily determine similarity in demographic and health indicators (as shown in figure 3) is a key consideration in ensuring representativeness in surveillance networks. Such information can be leveraged in the prioritisation of new sites. For instance, if costs and political instability prohibit establishment of a new site in a particular region, then it may be feasible to establish a site in a more stable and cost-effective location that displays very similar characteristics to ensure that the unstable region’s characteristics are represented in the wider network. Moreover, countries and regions without HDSS sites, but with similarities in factors associated with U5M, can draw on insights and learnings from sites in other countries and regions to tailor strategies and interventions.

In interpreting the results, it is important to account for limitations that exist in the data and analyses presented. The sample sizes of the DHS surveys used to quantify most of the U5M associated factors are insufficient to be representative at finer spatial scales than the administrative units used here. Thus, large area summaries are used, many of which mask local heterogeneities, as well as being spatial mismatches to the areas from which the HDSS sites draw their data in most cases. Moreover, while the set of demographic and health variables used in these analyses were shown to be strongly associated with U5M rates,^23^ this was not a perfect relationship, and multiple other relevant factors likely play a role, for which comprehensive and comparable data at subnational scales were not available. Recent DHS surveys were not available at the time of writing for those SSA countries outside of the 27 covered here, and, therefore, some HDSS sites and their contributions to the representativeness of the full set of sites in SSA could not be assessed. The inclusion of older or upcoming surveys, or data from alternative sources, could aid in filling these gaps to provide a more comprehensive picture. Finally, we have focused here on the networks of HDSS sites that continue to grow and be established, and how insights and learnings from these can be combined, compared and extended to subnational areas without such ongoing monitoring, rather than conducting a country-by-country assessment of how well the sites within their borders represent their own situation and variabilities. This may be a valuable area for future work, given country-specific objectives for SDG attainment.

Beyond U5M, the 2013–2016 West Africa Ebola epidemic and continued emergence of familiar and unfamiliar pathogens highlight the need for comprehensive health surveillance across a wide range of geographies to catch outbreaks before they become epidemics.^40^ HDSS sites have a key role to play in this area too, and the approaches and results presented here provide quantitative measures of the breadth of surveillance capacity across much of SSA. There is a need to ensure that the expansion of monitoring does not occur all in the same types of geographies, demographics and environments, leaving blind spots in our understanding and monitoring of mortality, health and disease.
