# Supplementary material for: Geographic coverage of demographic surveillance systems for characterising the drivers of childhood mortality in sub-Saharan Africa
Source: BMJ Glob Health. 2018 Apr 9;3(2):e000611. doi: 10.1136/bmjgh-2017-000611 (PMC5898321; doi:10.1136/bmjgh-2017-000611)
Supplement: Supplementary data [file bmjgh-2017-000611supp001.pdf]

The geographic coverage of demographic surveillance systems for characterising the drivers of childhood mortality in sub-Saharan Africa

Utazi CE[1,2], Sahu S[2], Atkinson PM[3], Tejedor-Garavito N[1,4,5], Lloyd CT[1] and Tatem AJ[1,5]

[1]WorldPop, Department of Geography and Environment, University of Southampton, Southampton, UK
[2]Southampton Statistical Sciences Research Institute, University of Southampton, Southampton, UK
[3]Faculty of Science and Technology, Lancaster University, Lancaster, UK
[4]GeoData, University of Southampton, Southampton, UK
[5]Flowminder Foundation, Stockholm, Sweden

Supplementary materials

Bayesian clustering of the subnational areas and coverage of the clusters

To group the subnational areas into clusters, we used the Bayesian central clustering methodology of [1]. A Bayesian finite Gaussian mixture model with different parameterizations was fitted to the 255 x 7 data matrix containing the estimates of the mortality indicators (7) for all the subnational areas (255). Markov Chain Monte Carlo (MCMC) techniques were used in the Bayesian framework to estimate the parameters of the model. The best model parameterization and number of clusters were chosen using a modified version of the Bayesian Information Criterion (BIC). As part of the methodology, a "central clustering" procedure was applied to clusterings of the best parameterization and number of clusters to obtain the central clustering (i.e. the clustering that is most representative of all the clusterings obtained from the MCMC iterations). Finally, the uncertainty associated with the clusterings was obtained as the probabilities with which the subnational areas were assigned to their clusters calculated as the frequency of being in the given cluster of the central clustering divided by the total number of MCMC iterations. Other details of the methodology are as reported in Utazi et al.[1].

Additional tables and figures

Table S1: Details of HDSS sites used in the study

Country	Original HDSS site name	Short name used	Organization	Network	Latitude	Longitude
Burkina Faso	Kaya	Kaya	Institut de recherche en Sciences de la Santé/Centre National de la recherche Scientifique et technologique (IRSS/CNRST)	INDEPTH	13.087310	-1.078599
Burkina Faso	Nanoro	Nanoro	National institutes of medical research (IRSS)	INDEPTH	12.689095	-2.191770
Burkina Faso	Nouna	Nouna	Le Centre de Recherche en Sante de Nouna	INDEPTH	12.741307	-3.866240
Burkina Faso	Sapone	Sapone	Centre National de Recherche et de Formation Sur le Paludisme	INDEPTH	11.993443	-1.283327
Burkina Faso	Ouagadougou	Ouagadougou ISSP	Institut Superieur des Sciences de la Population, Universite´ de Ouagadougou	INDEPTH	12.367252	-1.528928
Burkina Faso	Ouagadougou	Ouagadougou YH	Yedalgo Hospital	-	12.384482	-1.506943
Burkina Faso	Ouagadougou	Ouagadougou CGH	Charles de Gaulle Hospital	-	12.374301	-1.471578
Burkina Faso	Ouagadougou	Ouagadougou PK	Polesgo/Kossodo	-	12.399340	-1.567767
Cote d'Ivoire	Taabo	Taabo	Centre Suisse de Recherches Scientifiques en Cote D'Ivoire	INDEPTH	6.233803	-5.155671
Ethiopia	Butajira	Butajira	Butajira Rural Health Project	INDEPTH	8.111207	38.380628
Ethiopia	Dabat	Dabat	Dabat Research Center/University of Gondar	INDEPTH	13.167287	37.666635
Ethiopia	Gilbel Gibe	Gilbel_Gibe	Jimma University	INDEPTH	7.425300	37.115300
Ethiopia	Kersa	Kersa	Haramaya University	INDEPTH	9.589447	41.872284
Ethiopia	Kiltie Awlaeelo	Kiltie	Mekelle University	INDEPTH	14.273950	39.462200

		Awlaeelo				
Ethiopia	Addis Ababa	Addis Ababa BLSH	Black Lion Specialized Hospital	-	9.020144	38.749581
Ethiopia	Addis Ababa	Addis Ababa SPH	St. Paul Hospital	-	9.047699	38.728089
Gambia	Basse	Basse	Medical Research Council	GEMS/PERCH	13.311620	-14.219549
Gambia	Farafenni	Farafenni	Medical Research Council	INDEPTH	13.573665	-15.595471
Gambia	West Kiang	West Kiang	Medical Research Council	-	13.385618	-15.905948
Ghana	Ahafo Mining Area	Ahafo MA	Ghana Health Service	-	7.031380	-2.363100
Ghana	Dodowa	Dodowa	Ghana Health Service/Dodowa Health Research Centre	INDEPTH	5.881996	-0.098095
Ghana	Kintampo	Kintampo	Kintampo Health Research Centre	INDEPTH	8.043840	-1.727371
Ghana	Navrongo	Navrongo	Navrongo Health Research Centre	INDEPTH	10.846692	-1.334626
Ghana	Agogo	Agogo		-	6.794391	-1.071250
Ghana	Kumasi	Kumasi	Komfo Anokye Teaching Hospital in Kumasi	-	6.697519	-1.629198
Kenya	Kilifi	Kilifi	Kenya Medical Research Institute (KEMRI)-Wellcome Trust Research Programme	INDEPTH /GEMS /PERCH	-3.630607	39.850071
Kenya	Kisumu	Kisumu	KEMRI/Centre for Disease Control (CDC) HDSS	INDEPTH	-0.090014	34.770763
Kenya	Kombewa	Kombewa	Walter Reed/KEMRI	INDEPTH	-0.100000	34.516667
Kenya	Kwale-Kinango	Kwale Kinango	Institute of Tropical Medicine, Nagasaki University/NUITM-KEMRI Project	-	-4.175900	39.454590
Kenya	MBITA	MBITA	Institute of Tropical Medicine, Kenya Medical Research Institute, and Spring of Hope Project	INDEPTH	-0.435639	34.208682

Kenya	Webuye	Webuye	Moi University (Kenya) - VLIRUOS (Belgium) collaborative	-	0.616760	34.766550
Kenya	Nairobi	Nairobi APHRC	African Population and Health Research Center	INDEPTH	-1.243986	36.762660
Kenya	Nairobi	Nairobi KNHMDH	Kenyatta National Hospital & Mbaghati District Hospital	-	-1.300841	36.807473
Kenya	Siaya County	Siaya County	St. Elizabeth Lwak Mission Hospital	-	-0.130639	34.349487
Kenya	Western Kenya	Western Kenya	CDC	-	-0.094473	34.275064
Malawi	Karonga	Karonga	LSHTM/Malawi Epidemiology and Intervention Research Unit (MEIRU)	INDEPTH	-9.934499	33.936350
Malawi	Blantyre	Blantyre	Liverpool Wellcome Trust	-	-15.802993	35.021510
Mali	Bamako	Bamako CRHSP	Health Services Project (CRHSP)	GEMS	12.651464	-7.995804
Mali	Bamako	Bamako UoM	University of Maryland	PERCH	12.651218	-7.995655
Mali	Bamako	Bamako CVD	Center for Vaccine Development	-	12.621454	-8.028071
Mali	Bandiagara	Bandiagara	University of Maryland	-	14.350047	-3.611230
Mozambique	Chokwe	Chokwe	Chókwè Health Research and Training Centre (CITSC)	INDEPTH	-24.531315	32.998282
Mozambique	Manhica	Manhica	Institute for Global Health (ISGlobal)	INDEPTH /GEMS	-25.406745	32.806259
Nigeria	Nahuche	Nahuche	Zamfara State Ministry of Health	INDEPTH	11.783330	6.333335
Nigeria	Cross River (CRHDSS)	Cross River HDSS	University of Calabar	INDEPTH	4.965877	8.319807
Nigeria	Oriade	Oriade	University of Ife	-	7.517785	4.526348
Senegal	Bandafassi	Bandafassi		INDEPTH	12.5386	-12.3097
Senegal	Keur Soce	Keur Soce	University Cheikh Anta Diop-Department of Parasitology	-	13.9878	-16.0596

Senegal	Mlomp	Mlomp		INDEPTH	12.5173	-12.3366
Senegal	Niakhar	Niakhar	US 009 Suivi démographique, épidémiologique et environnemental, Niakhar	INDEPTH	14.3404	-16.4064
Tanzania	Ifakara	Ifakara	Ifakara Health Institute	-	-7.3208	36.9460
Tanzania	Korogwe	Korogwe	National Institute of Medical Research, Tanga Research Centre	-	-5.1559	38.4507
Tanzania	Magu	Magu	Tanzania-Netherlands Project to Support AIDS	-	-2.5920	33.4489
Tanzania	Rufiji	Rufiji	Future Health Systems project	-	-8.0979	38.3897
Tanzania	Bagamoyo	Bagamoyo	Ifakara Health Institute (IHI)	-	-6.4374	38.9078
Tanzania	Moshi	Moshi	Kilimanjaro Clinical Research Institute	-	-3.3200	37.3273
Tanzania	Pemba	Pemba	Johns Hopkins University	-	-5.2469	39.7813
Uganda	Awach	Awach	ENRECA-Gulu University Project	-	2.9702	32.4001
Uganda	Gulu	Gulu	ENRECA-Gulu University Project		2.7857	32.2858
Uganda	Iganga/Mayuge	Iganga Mayuge	Institute of Public Health Makerere University	INDEPTH	0.6134	33.4936
Uganda	Kalungu	Kalungu	-	-	-0.0692	31.8642
Uganda	Rakai	Rakai	The Rakai Health Sciences Program	INDEPTH	-0.7098	31.4056
Uganda	Kyamulibwa	Kyamulibwa	Uganda Virus Research Institute	INDEPTH	-0.3296	31.7353
Uganda	Toro	Toro	University of California, San Francisco (UCSF)	-	0.6553	30.2813
Zambia	Lusaka	Lusaka	Boston University at the University Teaching Hospital of Lusaka	PERCH	-15.4320	28.3148

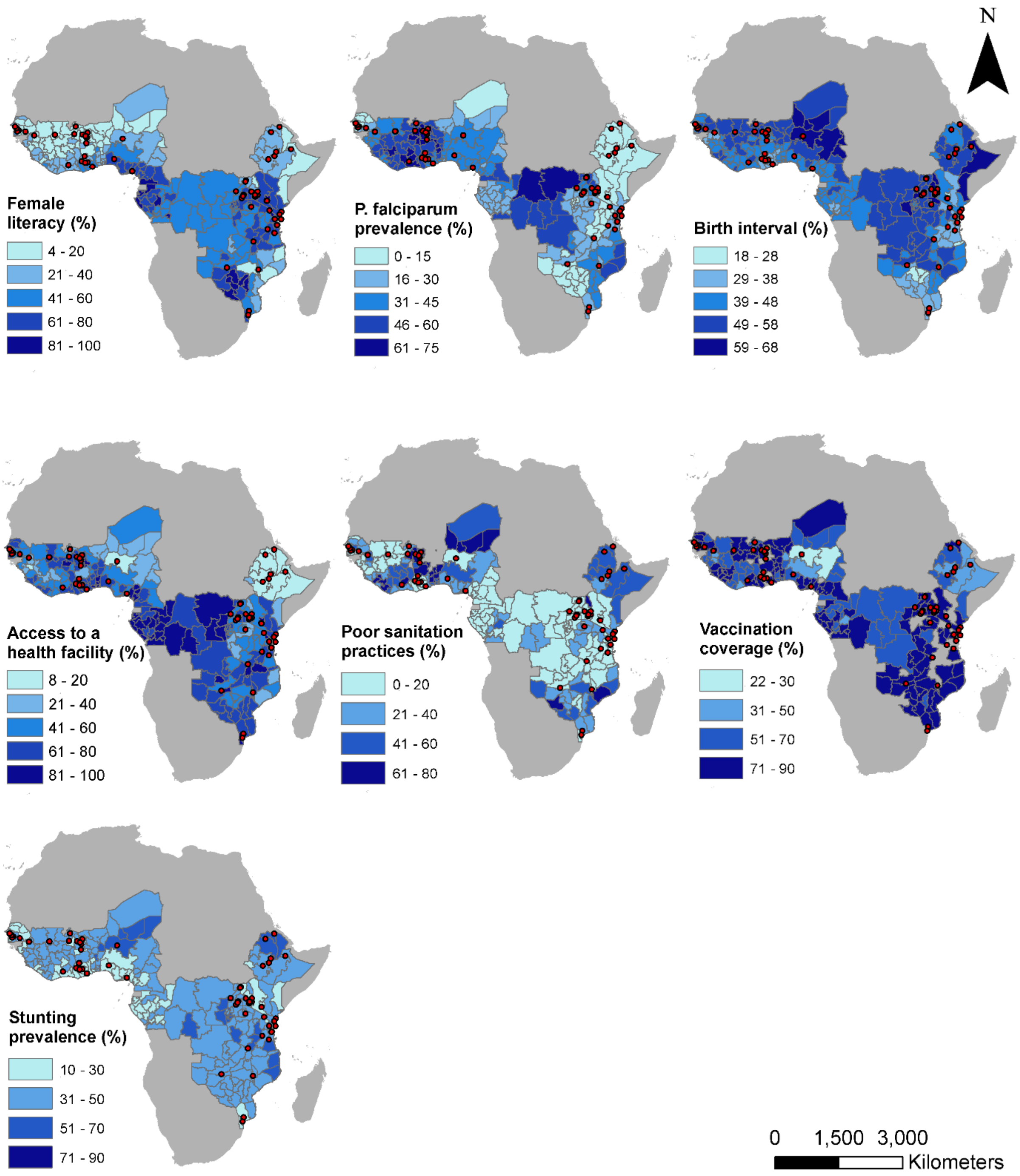

Figure S1: Maps of under-5 mortality indicators used in the study. The red filled circles are the locations of the HDSS sites.

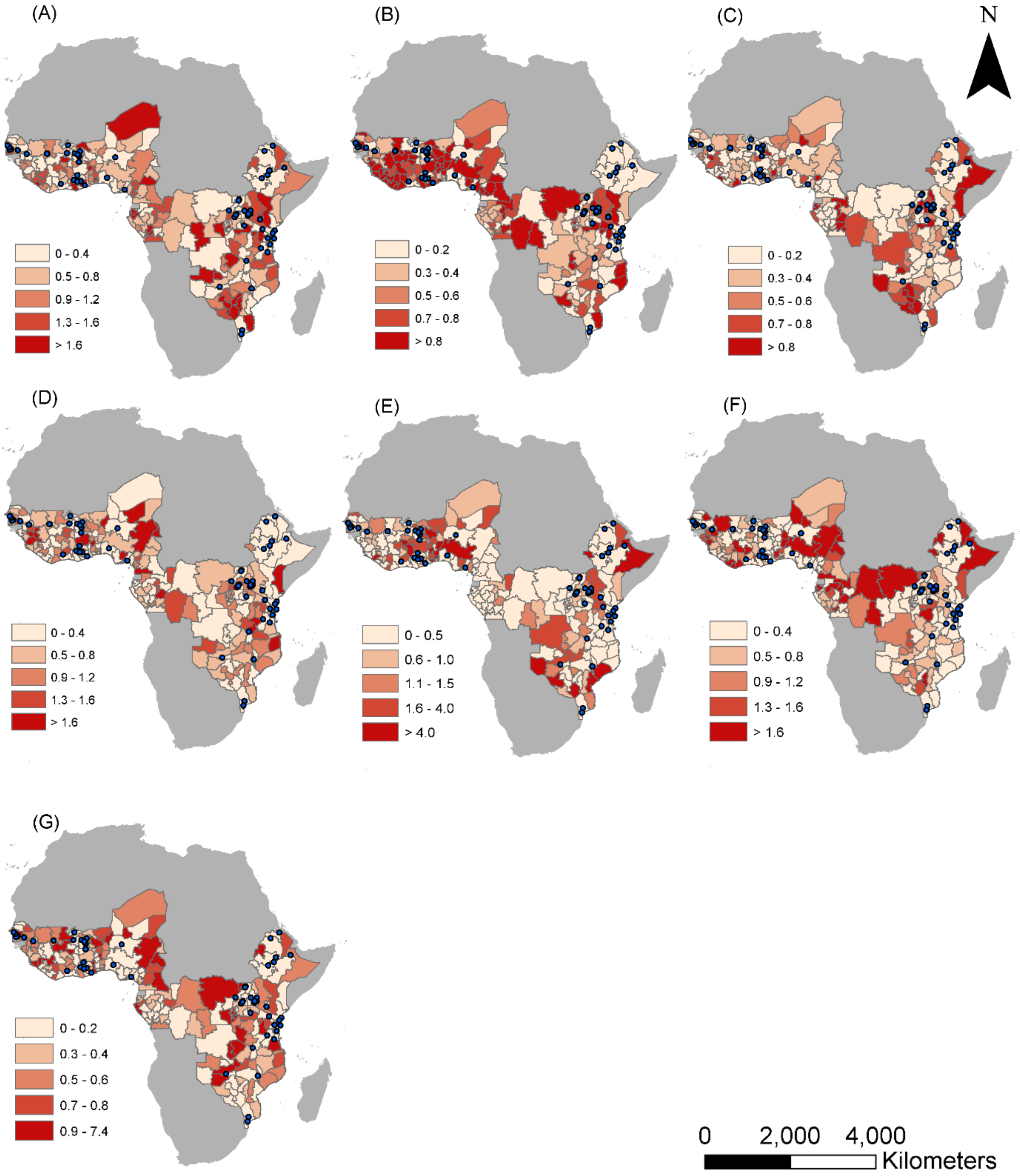

Figure S2: Euclidean distances from the HDSS sites for individual indicators. (A) Female literacy, (B) P. *falciparum* prevalence, (C) Birth interval, (D) Access to a health facility, (E) Poor sanitation practices, (F) Measles vaccination, (G) Stunting prevalence. The blue filled circles are the locations of the HDSS sites.

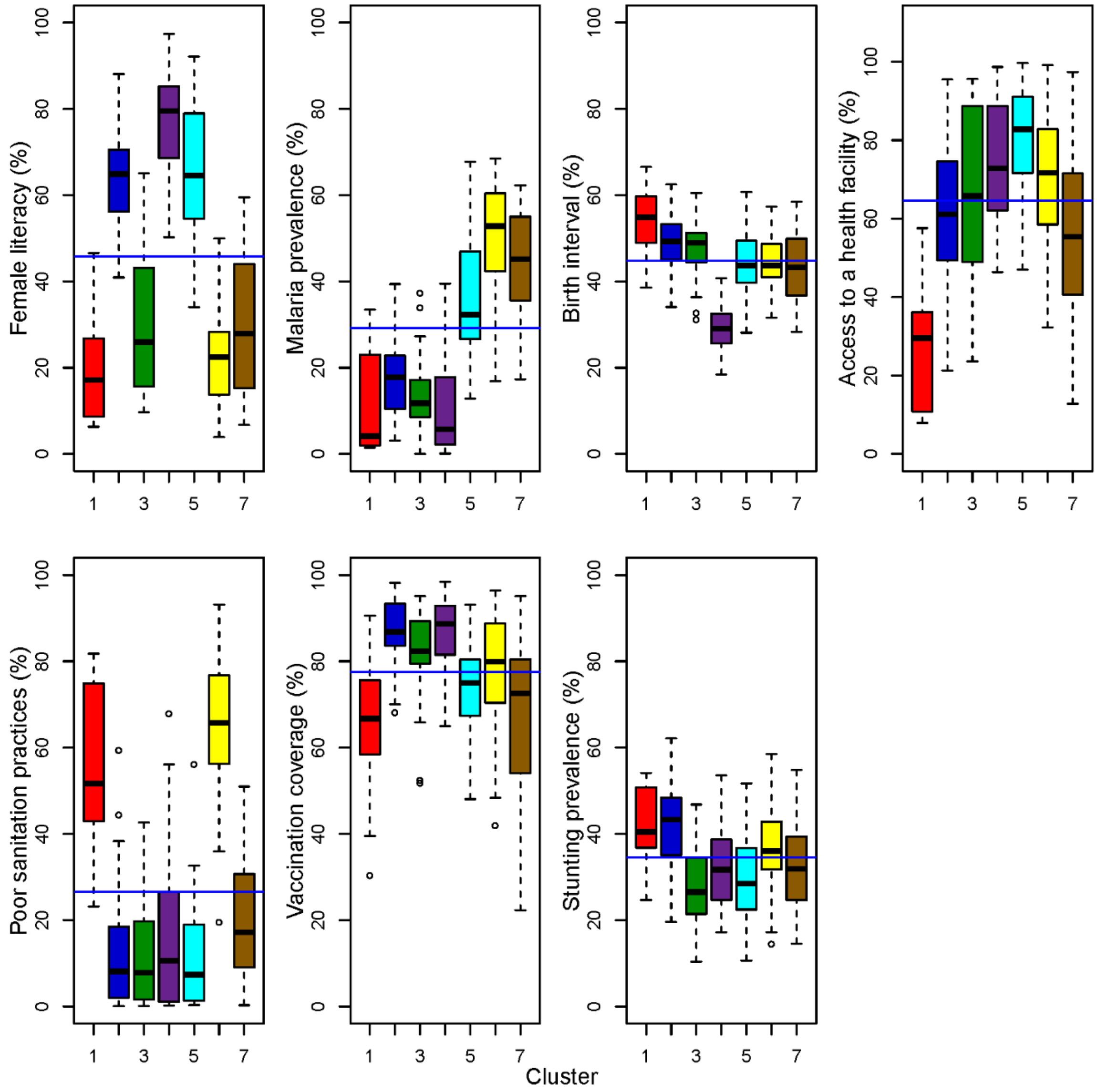

Figure S3: Distributions of under-5 mortality indicators in the clusters of the subnational areas in Figure 2. The clusters are coloured as in the figure. The blue lines are the means of the indicators.

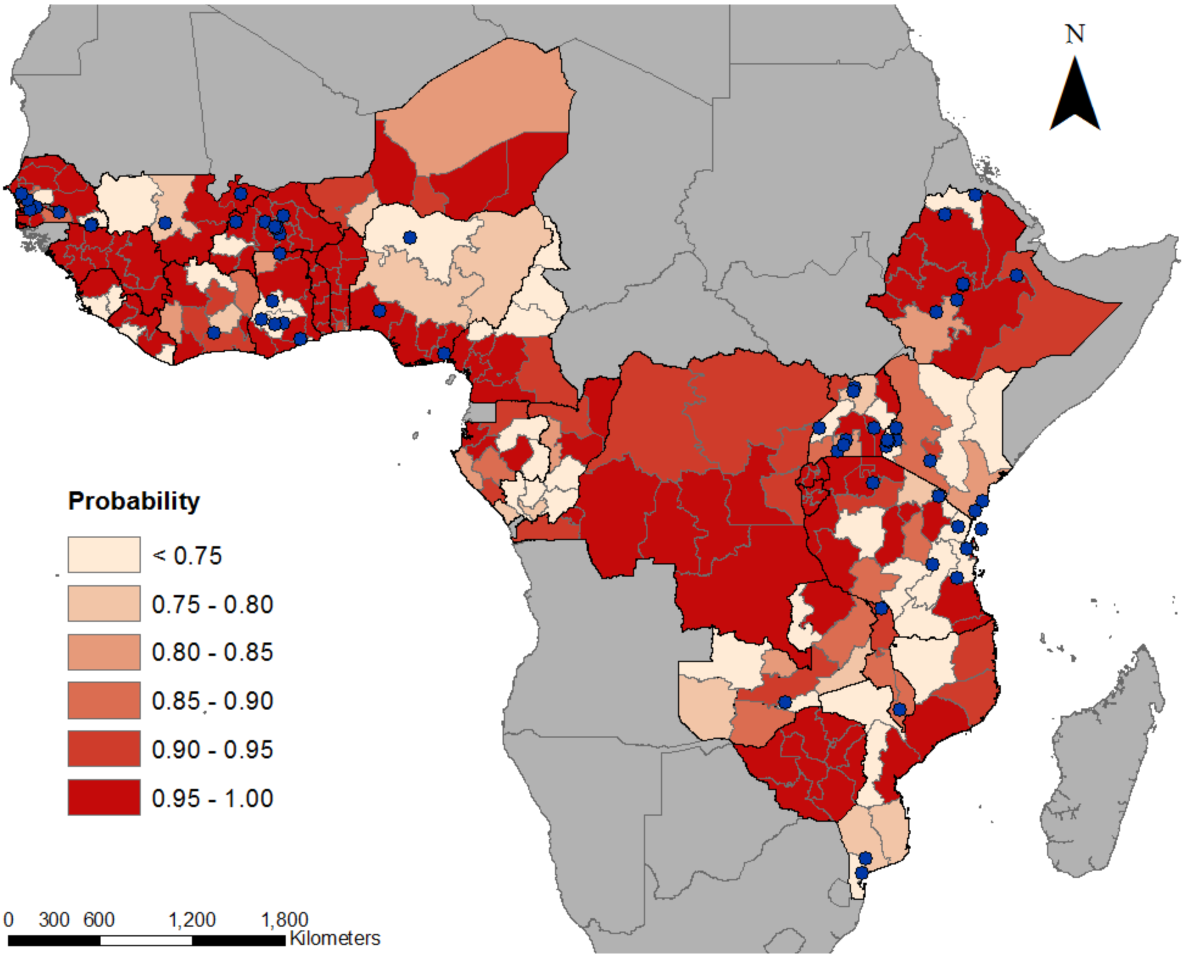

Figure S4: Uncertainty map for the clustering. Plotted are the probabilities of membership of the clusters.

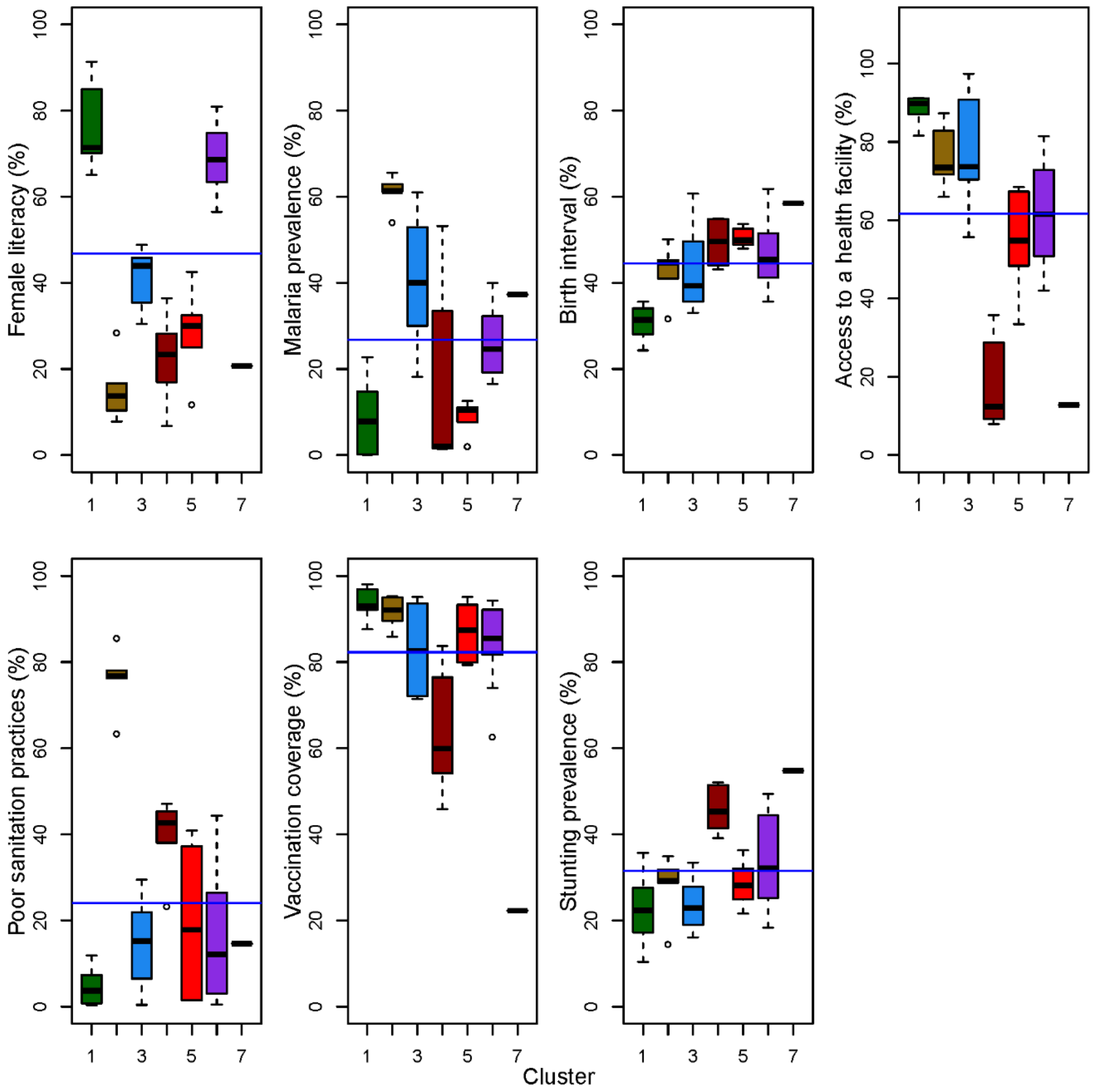

Figure S5: Distributions of under-5 mortality indicators in the clusters of the HDSS sites shown in the dendrogram in Figure 3. The clusters are coloured as in the figure. The blue lines are the means of the indicators.

References

1. Utazi CE, Sahu SK, Atkinson PM, Tejedor N, Tatem AJ. A probabilistic predictive Bayesian approach for determining the representativeness of health and demographic surveillance networks. Spatial Statistics 2016;17:161-78.
